# Online and Recovery-Oriented Support Groups Facilitated by Peer Support Workers in Times of COVID-19: Protocol for a Feasibility Pre-Post Study

**DOI:** 10.2196/22500

**Published:** 2020-12-18

**Authors:** Jean-Francois Pelletier, Janie Houle, Marie-Hélène Goulet, Robert-Paul Juster, Charles-Édouard Giguère, Jonathan Bordet, Isabelle Hénault, Alain Lesage, Luigi De Benedictis, Frédéric Denis, Roger Ng

**Affiliations:** 1 Department of Psychiatry and Addictology Montreal Mental Health University Institute – Research Centre University of Montreal Montreal, QC Canada; 2 Department of Psychiatry Yale University New Haven, CT United States; 3 Department of Psychology Université du Québec à Montréal Montreal, QC Canada; 4 School of Nursing University of Montreal Montreal, QC Canada; 5 Montreal Mental Health University Institute – Research Centre Montreal, QC Canada; 6 Quebec Association of Peer Support Workers Montreal, QC Canada; 7 Université de Tours Tours France; 8 Kowloon Hospital Hong Kong China

**Keywords:** peer support workers, internet-based peer support groups, personal-civic recovery, clinical recovery, COVID-19 Stress Scales, peer support, feasibility, mental health, COVID-19, intervention, recovery

## Abstract

**Background:**

In times of pandemics, social distancing, isolation, and quarantine have precipitated depression, anxiety, and substance misuse. Scientific literature suggests that patients living with mental health problems or illnesses (MHPIs) who interact with peer support workers (PSWs) experience not only the empathy and connectedness that comes from similar life experiences but also feel hope in the possibility of recovery. So far, it is the effect of mental health teams or programs with PSWs that has been evaluated.

**Objective:**

This paper presents the protocol for a web-based intervention facilitated by PSWs. The five principal research questions are whether this intervention will have an impact in terms of (Q1) personal-civic recovery and (Q2) clinical recovery, (Q3) how these recovery potentials can be impacted by the COVID-19 pandemic, (Q4) how the lived experience of persons in recovery can be mobilized to cope with such a situation, and (Q5) how sex and gender considerations can be taken into account for the pairing of PSWs with service users beyond considerations based solely on psychiatric diagnoses or specific MHPIs. This will help us assess the impact of PSWs in this setting.

**Methods:**

PSWs will lead a typical informal peer support group within the larger context of online peer support groups, focusing on personal-civic recovery. They will be scripted with a fixed, predetermined duration (a series of 10 weekly 90-minute online workshops). There will be 2 experimental subgroups—patients diagnosed with (1) psychotic disorders (n=10) and (2) anxiety or mood disorders (n=10)—compared to a control group (n=10). Random assignment to the intervention and control arms will be conducted using a 2:1 ratio. Several instruments will be used to assess clinical recovery (eg, the Recovery Assessment Scale, the Citizenship Measure questionnaire). The COVID-19 Stress Scales will be used to assess effects in terms of clinical recovery and stress- or anxiety-related responses to COVID-19. Changes will be compared between groups from baseline to endpoint in the intervention and control groups using the Student paired sample t test.

**Results:**

This pilot study was funded in March 2020. The protocol was approved on June 16, 2020, by the Research Ethics Committees of the Montreal Mental Health University Institute. Recruitment took place during the months of July and August, and results are expected in December 2020.

**Conclusions:**

Study results will provide reliable evidence on the effectiveness of a web-based intervention provided by PSWs. The investigators, alongside key decision makers and patient partners, will ensure knowledge translation throughout, and our massive open online course (MOOC), *The Fundamentals of Recovery*, will be updated with the evidence and new knowledge generated by this feasibility study.

**Trial Registration:**

ClinicalTrials.gov NCT04445324; https://clinicaltrials.gov/ct2/show/NCT04445324

**International Registered Report Identifier (IRRID):**

PRR1-10.2196/22500

## Introduction

### Need for a Feasibility Trial

In recent pandemics, social distancing, isolation, and quarantine have precipitated depression and anxiety [1,2]. It is expected that isolation and physical (social) distancing due to the COVID-19 pandemic will lead to similar consequences as confined people are detached from their loved ones, deprived of personal liberties, and devoid of purpose owing to altered routine and livelihood. These consequences will probably be amplified by the economic recession and the ensuing increase in unemployment and poverty. Moreover, those with pre-existing mental illness might suffer from limiting interpersonal interactions that are central to their management, as well as reduced access to helpful but “nonessential” (and thus often canceled) psychiatric services [3]. Successful use of quarantine and distancing as a public health measure requires us to reduce, as much as possible, the negative effects associated with it [4].

In response to this situation, this feasibility study of a trial offers a transitional measure of online social support for people suffering from (1) psychotic disorders or (2) anxiety or mood disorders, and to assess its effects in terms of both personal-civic recovery and clinical recovery. Transitional peer support groups will be organized and led by trained peer support workers (PSWs). PSWs are persons with first-hand lived experience of mental health problems or illnesses and who are further along in their own recovery journey. Upon training, they can provide supportive services when hired to fill such a paid specialty position, as now recommended by recovery-oriented best practices guidelines [5,6]. Indeed, recovery focuses on how individuals can have more active control over their lives (agency) [7]. It is characterized by a search for the person’s strengths and capacities, satisfying and meaningful social roles, and mobilizing formal and informal support systems. Peer support has thus become one predominant feature of the recovery paradigm and, as per their title and function, PSWs specialize in peer support.

#### A Problem to Be Addressed: Measuring the Effects of PSWs on Recovery

In Canada, the incremental economic burden of mental health problems or illnesses (MHPIs), which incorporates the use of medical resources and productivity losses due to long-term and short-term disability, as well as reductions in health-related quality of life, is estimated to be more than $50 billion per year. By calculating all health services utilization, long-term and short-term work loss, and health-related quality of life and their dollar valuations, Lim et al [8] have concluded that over one-half was due to reductions in health-related quality of life. Decreasing pain and suffering, overcoming disability due to MHPIs, and helping Canadians affected by MHPIs to recover, instead of solely treating them for their MHPIs, is a major public health issue, even more so in times of isolation and quarantine in the midst of a pandemic since social support is known to be an important factor that can affect mental health [9]. This is why, along with other sociopolitical or historical considerations, recovery is now the official leading paradigm in the transformation of mental health systems and policies in Canada [10], as is also the case in the United States [11], the United Kingdom [12], and elsewhere in the world, and as promoted by the World Health Organization (WHO) [13].

As a social movement echoing the historical claims of other social movements since the 1960s and 1970s, including the antipsychiatry movement, the origins of recovery in mental health are now fairly well documented [14,15]. Yet, tensions persist about the meaning and ownership of recovery [16,17]. Generally speaking, there are two major portrayals of recovery [18,19]. One is akin to the notion of cure in the field of physical health; *clinical* recovery refers primarily to the reduction of psychiatric symptoms through a curative approach to the disease using psychopharmacology and psychotherapy. With this first axiom of recovery, the role of the ill person is mainly to follow the instructions of professionals and comply with prescribed treatments. On the other hand, a more *personal-civic* axiom of recovery promotes the empowerment of the persons, their ownership and authorship of their own history, their autonomy, and their independence in living free from any labeling diagnosis [20]. Recovery is not the disappearance or the eradication of symptoms but a redefinition of oneself in light of lived experience as a person who managed to live with an MHPI, and who found a new balance in life toward well-being and quality of life, with or without enduring psychiatric symptoms or treatment.

The experience of living in recovery is particularly useful for sharing among peers who are coping, and/or have coped, with similar issues. The commonality is the struggle and emotional pain that can accompany the feeling of loss and/or hopelessness due to the consequences of MHPIs, rather than in relation to a specific symptom or psychiatric diagnosis. This commonality might also be in relation to sex and gender considerations [21,22], more than in relation to specific conditions. Peer-to-peer communication is a widespread phenomenon (eg, in groups like Alcoholics Anonymous [23] or Al-Anon for families [24]). Access to PSWs has been widely advocated internationally by service user researchers [25,26] and by professional organizations [27,28]. Provision of peer support has been identified as a fidelity requirement for recovery-orientated services [11,29] where the PSWs bring a focus on health, recovery, and quality of life, rather than on illness and disability [30]. They provide the mental health service users a validation of their lived experience and unique experiential knowledge for facilitating the reclaiming of their lives as full members of the community and for remaining so [31]. The Mental Health Commission of Canada emphasizes that patients living with MHPIs who interact with PSWs “will not only feel the empathy and connectedness that comes from similar life experiences, but that this interaction also fosters hope in the possibility of a recovery that includes health, wellbeing, quality of life, and resilience” [32]. Since 2005, recovery and PSWs have also been at the core of Mental Health Action Plans in the province of Quebec [33,34]. Yet, we still do not know how a recovery-oriented mental health organization that would include PSWs as staff members would improve recovery from the perspective of persons living with MHPIs.

#### Principal Research Questions

The principal research questions are whether our novel PSWs-facilitated online intervention will have an impact in terms of (Q1) clinical recovery potential and (Q2) personal-civic recovery potential. We also question (Q3) how these potentials can be impacted by the COVID-19 pandemic and (Q4) how the lived experience of people in recovery can be mobilized to cope with the situation. Finally, we also explore (Q5) how sex and gender considerations can be taken into account for the pairing of PSWs with service users beyond considerations based solely on psychiatric diagnoses or specific MHPIs [35]. We aim to collect data for a future randomized controlled trial (RCT) [36] by clarifying a certain number of remaining uncertainties and by detecting an effect that would be specifically attributable to transitional peer support as facilitated by trained PSWs. We will thus consider scientific reasons, processes, resources, and management in preparation for a more definitive trial. Indicators of feasibility will include recruitment rates practices, participants and facilitators, as well as feasibility and retention rates in the study protocol [37].

#### Why a Feasibility Study of a Trial Is Needed Now

The main problem that this feasibility study of a trial addresses is that the scientific literature on the attribution of a specific effect of paid PSWs, in terms of clinical and personal-civic recovery potentials among persons living with psychotic disorders and/or anxiety and mood disorders, is sparse. Indeed, although we already know that patients served by case management teams with PSWs have shown greater treatment engagement, more satisfaction with their life situation and finances, and fewer life problems than compared to case management alone [38], it is the effect of teams with PSWs that has been mostly evaluated. It is thus possible that such effects could be attributable to mental health professionals, other than PSWs, and who would have endorsed recovery as a guiding principle for their own professional practice. We may know who PSWs are, but we do not know much about what PSWs do that would be complementarily different to these professionals. This has the adverse potential of hindering the harmonious integration of PSWs into professional teams when their role is not well accepted or understood, which was sometimes the case, for instance, in France, and as suggested by Demailly (and Garnoussi), who observed a possible rejection phenomenon of PSWs [39-41].

##### Evidence From the Literature

In 2014, Lloyd-Evans et al [42] published a systematic review and meta-analysis of RCTs of peer support programs, defining peer support “as a way to promote recovery for anyone who has experienced mental ill health, irrespective of diagnosis.” Their systematic review and meta-analysis included trials intended for people with psychotic disorders. They found that programs varied in content, group, or individual delivery; face-to-face or internet-based delivery; degree of support from local mental health services; and extent of provider training. Such limitations make it difficult to recommend the practice on a scientific basis rather than as a response to the otherwise very legitimate social and political demands of the mental health service users’ movement. Several programs focused on individual self-management, as shown by Johnson et al [43], and Milton et al [44], who reported that 64 (29%) of 218 participants in the intervention versus 83 (38%) of 216 in the control group were readmitted to acute care within 1 year (odds ratio 0.66, 95% CI 0.43-0.99; P=.044). This “individualistic” approach to recovery and focus on readmission to acute care as a primary outcome has been criticized by some advocates of service users’ involvement in mental health research [45] and who see this as a practice of silencing and of masking the epistemic injustice [46] that people living with MHPIs have collectively and historically suffered in traditional psychiatry [47].

A quasi-experimental group design was used by Felton at al [48] to compare outcomes of patients with psychotic disorders (n=104). They reported that among these patients, those served by teams *with* PSWs demonstrated greater gains in several areas of quality of life, and overall reduction in the number of major life problems experienced. This might be due to team dynamics and culture, not necessarily to the supportive presence of PSWs toward these patients. Then, with regards to anxiety and mood disorders, Pfeifer and colleagues [49] also conducted a meta-analysis, examining the effect of peer support on clinical recovery (symptoms), but not on personal-civic recovery. They included professionally led peer support groups and found that voluntary peer support complementary interventions from nonprofessionals were superior to usual care in reducing depressive clinical symptoms. They also conclude that despite potential economic advantages and the multiple mechanisms through which these could help patients with anxiety and mood disorders, such programs have been limited in their availability and integration with formal mental health treatment. This is currently the case in the province of Quebec, where it is planned that there would be PSWs in teams dedicated to the treatment of psychotic disorders (but not in teams dedicated to treatment of anxiety or mood disorders). Psychotic disorders affect about 1% of the population. Studying the potential impact of PSW-led online group interventions for much more common disorders like anxiety and mood disorders, in conjunction with formal mental health treatment, could benefit 10 times more people. Indeed, Statistics Canada reports that 10.1% of Canadians ≥15 years of age declared symptoms that met the criteria for anxiety (4.7%) and depression (5.4%) [50].

##### Search for Existing Trials

As part of the development of a new training program for PSWs, a joint undergraduate program of the Department of Psychiatry and the Vice-Deanery for Health Sciences at the Faculty of Medicine of the University of Montreal, we systematically searched the literature for examples of evidence-based PSWs interventions we could replicate and train our students to conduct. A recovery-oriented group intervention by PSWs that we became familiar with over the years is the Citizenship Enhancement Project [51]. Derived initially from research on mental health outreach to persons who are homeless [52], this intervention was designed to address the specific community and social inclusion needs of persons with MHPIs and comorbid criminal justice histories, as well as to respond to the high rates of criminal recidivism for this population. Indeed, drawing from social science theories that propose social and civic participation as a measure of one’s involvement in society [53], our engagement framework [54] emphasizes the importance of opening up opportunities for participation to persons at risk of marginalization. An RCT was conducted in the United States in 2012 to compare outcomes for participants receiving this intervention, along with the usual public mental health services, to those receiving public mental health services alone. Analysis of baseline, 6-month, and 12-month interviews showed significantly reduced alcohol and drug use and significantly increased quality of life for the intervention group compared to the control group [55]. In close collaboration with the Yale Program for Recovery & Community Health that initiated the Citizenship Enhancement Project, with patient research partners we have successfully translated and transposed this model into the Projet Citoyen at the Institut universitaire en santé mentale de Montréal (IUSMM) [56]. In both places, the role of PSWs consisted of facilitating discussion groups on issues of social and civic participation. Three key differences make it necessary to further evaluate the effect of the PSWs’ group intervention specifically. Firstly, involvement in the Criminal Justice System within the past 2 years is an inclusion criterion for the former, not for the latter. Secondly, another inclusion criterion for the former is that it was intended for people living with psychotic disorders and concurrent substance abuse disorders, therefore not including people living with anxiety or mood disorders, as was the case with the Projet Citoyen. Thirdly, the Citizenship Enhancement Project and Projet Citoyen have been evaluated, but not in terms of personal-civic recovery with a pre-post research design. In brief, searches for existing meta-analysis of clinical trials for PSWs’ group intervention confirm that no previous randomized trial has compared the outcomes of a group intervention led by PSWs combined to formal mental health treatment in terms of clinical and personal-civic recovery for people living with psychotic disorders, and/or anxiety and mood disorders.

#### How Results Will Be Used

A result of PSWs’ online group intervention efficacy in terms of both clinical and personal-civic recovery potentials for people living with psychotic disorders, and/or anxiety and mood disorders, would privilege the role of PSWs in recovery-oriented peer-to-peer support groups. The study will impact the conceptualization of recovery- and citizenship-oriented mental health care, clinical training, and in mental health treatment resource allocation and for informing nonspecialized clinicians as well as the public. Indeed, an embedded observational and qualitative study performed with postgraduate students and patient research partners will improve the understanding of the experiential knowledge translation and knowledge sharing dynamics among participating patients living with MHPIs and among PSWs. Decision making will also be informed for the definition of a university mission for all psychiatric facilities of the University of Montreal’s Integrated University Health and Social Services Network (IUHSSC). This network advances the integration of the university mission of care, teaching, and research, by facilitating knowledge translation and technology assessment in order to improve access to evidence-based care. This is a vast integrated network of health and social services organizations and public establishments with a university vocation, including those psychiatric facilities where PSWs do their final internship before being eventually hired. The territory of this network represents 46% of the population of the province of Quebec and the three Integrated University Health and Social Services Centers affiliated with the University of Montreal are partners of this study. Since 2015, the IUHSSC organization is the gateway to the public service system where the Quebec population can turn in case of health problems and/or psychosocial problems, including MHPIs. Due to their university affiliation, their mission is to contribute to academic training as well as to the development and dissemination of scientific knowledge. The study will inform the PSWs’ training program by generating a better understanding of the specific effects attributable to the PSW group intervention.

#### Previous Works

In 1994, Daniel Fisher, a person with lived experience of several psychiatric hospitalizations prior to becoming a psychiatrist and renowned author and speaker, released an empowerment model of recovery based on the principles that emerged from the lived experience of persons living with MHPIs. Among those principles is *personhood*: “we are full human beings and deserve respect and full citizenship” [57]. More recently, Davidson and colleagues suggested that, as a sense of empowerment and control over one’s life emerges, people in recovery may start to demand the same rights and duties as other citizens [58,59]. Supporting people living with MHPIs in exercising their citizenship (which refers to personal confidence/hope, willingness to ask for help, goal/success orientation, reliance on others, and no domination by symptoms) might be a precondition for their recovery, not an eventual reward contingent on the person overcoming his/her disability first. Rowe and Davidson [60] have thus suggested that research on clinical recovery, often invoked to illustrate personal recovery’s different meaning and mission, also inspired the mental health community with its findings that people with chronic MHPIs often do “get better” in the traditional clinical sense. Personal recovery transferred the hope these findings gave to the conviction that people could recover a full and meaningful life even without achieving a clinical cure or remission. Rowe and Davidson [60] state:

Finally, recovering citizenship means that while recovery is replenishing its social roots, it also reminds citizenship, with its emphasis on the person’s rightful place in society, of the person’s unique journey to citizenship and life as a citizen.

Social interaction, which is essential to community membership, involves the development and maintenance of reciprocal relationships between members of a community, each of whom are, in principle, equal cocitizens to each other [52,61]. Thus the notion of collective citizenship, as suggested by Quinn et al [62] when applying this model to the domain of mental health, where people are often treated in individualistic ways, kept apart separately, and experience marginalization. The collectivistic dimension is imperative in promoting participation, empowerment, and social change for people living with MHPIs.

Indeed, the Mental Health Commission of Canada considers that “involvement within community” is an integral component of a definition of personal recovery. We thus combined the Recovery Assessment Scale [63] and the Citizenship Measure [64], because the former does not include any item on this civic participation dimension, as does the latter. We first translated these tools in French using a translation-back-translation method [65], then found statistically high convergent validity between them [66]. Patient research staff have administered these tools to 845 French-speaking IUSMM patients living with various MHPIs. We found statistically significant male-female differences, suggesting that males felt more independent and self-confident but also more isolated than females, especially in relation to the intention to seek and receive help. Such results in terms of sex differences [67,68] were discussed in class among PSW apprentices to inform and guide their future practice. The current proposal will allow us to further explore the influence of sex and gender in the pairing between PSWs and peer-supported service users. We questioned if this pairing could be favored by taking into account such sex-gender considerations, rather than solely on a diagnostic basis (eg, should a patient living with psychotic disorders only be paired to PSWs living with the exact same condition?).

A criterion for being recruited as a PSW will be to have successfully completed at least 180 hours of training within the microprogram (eg, undergraduate courses PST1000-Recovery & Global Health + PST1001-Ethics of Recovery for PSWs). We have published on the “reversed flipped class” approach in use in this 1-year-long microprogram where senior PSWs take turns as recovery experts and teaching partners, and by which patients/students learn from each other in a peer support–like atmosphere by reflecting on their lived experience of recovery [69-71]. This system, including the evaluation of the final internship in clinical environments affiliated with the University of Montreal, was adapted from the Canadian Medical Educational Directives for Specialists (CanMEDS). The CanMEDS are commonly used by medical boards in Canada and elsewhere to create competency-based medical training programs [72]. With permission from the Royal College of Physicians and Surgeons of Canada, we have adapted the CanMEDS for the training and supervision of our PSWs [73,74]. PSWs will be recruited and hired in the form of service contracts through the Association des pairs aidants du Québec because the job title of PSW is not yet recognized in the nomenclature of job titles, labels, rates, and salary scales of the health and social services network. Since they cannot hire them directly under this job title, several Quebec institutions like the IUHSSC organization use this mechanism to have PSWs in their mental health teams, as recommended in the governmental Mental Health Action Plan. The Association des pairs aidants du Québec is a company legally constituted under Part III of the Loi des companies du Québec. It is a social enterprise self-managed by and for PSWs and consumers (peer-run agency), grouped within a professional association in order to improve, promote, and trade their specific expertise and knowledge. Peer-run agencies are staffed and operated completely by self-described mental health consumers who provide services such as self-help, activity groups, and drop-in groups. Yanos et al [75], as well as Miyamoto and Sono [76], have shown that involvement in such services was associated with better community adjustment, the use of more coping strategies, and a greater proportion of problem-centered coping strategies. PSWs recruited by job posting to facilitate transitional peer support groups will not themselves be participants in this study. However, they will have a specific mandate because they will generate certain data analyzed in the context of this study, in particular with regard to the recognition, valorization, and use of the experiential knowledge of persons in recovery, particularly, but not limited to, to their response and coping strategies under the COVID-19 pandemic.

## Methods

### Proposed Feasibility Trial

#### Design

The “signatures” of MHPIs is a term formulated by the American National Institute of Mental Health to designate the broad range of genetic, biological, psychological, and social factors that may “sign” a specific mental disorder, depending on an individual’s sex, history, lifestyle habits, etc [77]. In 2010, based on the recommendations of an international advisory committee composed of some of the best scientists in the world in the field of psychiatric research, the Research Centre of IUSMM implemented the “Signature Bank” project for the collection of biological and psychosocial dimensional signatures from all psychiatric emergency patients of the IUSMM (catchment area of about 600,000 inhabitants). More than 4000 patients are treated annually at the IUSMM, while an additional 2000 patients per year are treated by means of outpatient or ambulatory services. Our activities provide us with one of the largest populations of patients with MHPIs in Canada. What is unique about this ambitious longitudinal research project is the extensive involvement of the IUSMM hospital site in the attempt to establish an exclusive niche for discoveries in the signatures of MHPIs. By collaborating with the Research Centre, IUSMM hospital managers have contributed to the implementation of this large-scale project that aims to measure the (epi)genetic, biological, psychological, and social signatures of people living with MHPIs who receive the IUSMM’s clinical services. Typically, these measures are obtained at four different points in the clinical visit of patients at the IUSMM: when patients are admitted to the psychiatric emergency services (T1), when they are discharged from the hospital (T2), when they are admitted to an outpatient clinic (T3), and 12 months after T3 (T4) [78]. With this proposal we go even further in understanding not only the signatures of MHPIs but also the dimensions of personal-civic recovery, as reported by our patients who will additionally complete the Recovery Assessment Scale and Citizenship Measure components.

#### Planned Trial Interventions

##### Control Intervention

When a person shows up at the Emergency Department of IUSMM for the first time, he or she is systematically approached by a research nurse after a first medical authorization is granted for that person to be approached (sometimes this authorization is not granted for medical or security reasons). The research nurse then explains the objectives of the Signature Bank project and invites the person to participate. Those who agree to participate sign the Information and Consent Form (T1), fill out a series of questionnaires, including ones on sociodemographics; consent to the taking of biological samples; and asked if they are willing to be contacted for other research purposes (like our own study). Then, as with any other IUSMM patients, they are evaluated by the Evaluation and Liaison Module during their hospital stay. A diagnostic is established or confirmed by ward psychiatrists and coded according to the WHO International Classification of Disease–10th Revision (ICD-10) [79]. Based on the diagnosis (or diagnoses), after discharge (T2), they are referred to a specialized outpatient clinic (T3). Whether for psychotic disorders or for anxiety and mood disorders, pharmacotherapy, psychotherapy, or a combination of both are then offered in accordance with the guidelines of the Royal College of Physicians and Surgeons of Canada. Of the Signature Bank participants diagnosed with psychotic disorders, or anxiety or mood disorders, and who consent to participate in our study, half will receive only the control intervention, while the other half will also receive our experimental PSW-led online group intervention (random allocation control/experimental intervention ratio=2:1).

Inclusion criteria include patients recruited from the Signature Bank data collection project diagnosed with (1) schizophrenia and psychotic disorders (ICD F20-F29), or with (2) anxiety or mood disorders (ICD F30-F49), (3) aged 18 years old or more, and (4) who have already consented to be contacted by telephone to be invited by our team to participate in this pilot study. Exclusion criteria include (1) active suicidal intentions, (2) marked cognitive impairment, and (3) no access to an electronic device with a webcam and microphone to participate in the online transitional peer support group.

##### Experimental Intervention

Trained PSWs will learn with participants via a series of 10 colearning workshops that they will organize and facilitate as focus group panels in a manner to simulate a typical peer support group [80]. The difference between our experimental and transitional online peer support groups and real community-based peer support groups is that (1) they will be facilitated by trained PSWs; (2) they will have a personal-civic recovery focus; and (3) they will have a fixed, predetermined duration (a series of 10 weekly 90-minute online workshops). Indeed, as defined by the WHO [81]:

Peer support groups bring together people who have similar concerns so they can explore solutions to overcome shared challenges and feel supported by others who have had similar experiences and who may better understand each other’s situation. Peer support groups may be considered by group members as alternatives to, or complementary to, traditional mental health services. They are run by members for members so the priorities are directly based on their needs and preferences. Peer support groups should ideally be independent from mental health and social services, although some services may facilitate and encourage the creation of peer support groups.

The objective is to prevent the deterioration, in times of pandemic, of the participants’ recovery potential. It is also a question of stimulating this potential by encouraging them to share their worries and their coping strategies in relation to the current acute situation. More generally, they will be asked to project themselves beyond this situation and to discuss future challenges of inclusion and social participation (eg, by attending already existing peer support groups) in the short or long term, and of which they will have become aware of during the intervention. This is why this intervention is considered to be transitional. Their own goals during the pandemic may be different from those post pandemic, and the effects of the response may also be different. However, similar to Taylor et al [82] who developed the COVID-19 Stress Scales, which we will use (see the *Outcome Measures at Follow-up* section), the whole intervention is intentionally designed to be readily adaptable to other (pandemic) situations.

To generate a collective narrative [83-85], the output of each workshop will have a brief written account of the group discussion, upon which the next workshop will open, and so on. To trigger discussion, PSWs will use animation cards and techniques inspired and adapted from the Malette COMETE toolkit, which was developed in France to help health care teams develop the psychosocial skills of patients in therapeutic education. These cards are available for free [86]. Each workshop will be filmed via the Zoom secured video communication system for subsequent qualitative observational and content analyses. In accordance with our model of patient engagement [87,88], PSWs will start every time by disclosing being themselves persons in recovery, and feed with content drawn from their lived experience while asking participants to share their own lived experience and coping strategies. This is in line with experiential learning [89]. After each workshop, the PSWs will meet for a 30-minute debriefing session, asking themselves what they learned, personally and professionally (also recorded). Christens [90] has conceptualized the relational process of recovery mentorship as an expression of psychological empowerment, as embodied in and practiced by the PSWs as mentors, and as an egalitarian relationship that helps facilitate the empowerment of the mentees. There are no reports of PSW-led online group intervention–related adverse reactions, but participants will be monitored for any contraindications and adverse events.

#### Allocation

Upon reception of the signed Information and Consent forms by email, consecutive referrals will be randomly allocated by computer algorithm to one of the two modalities at point of entry into the study on acceptation into the protocol using a computerized program (eg, randomization.com). At this entry point, the participant will have been evaluated, diagnosed, met inclusion criteria, and given formal consent for the randomization procedure. To ensure a balance in the allocation for the strata and thus control the risk of a secular trend in the composition of groups, random block sizes in a random order will be used (3, 6, 9, etc). Participants will thus be randomly allocated to the trial arm (n=20, 2 groups of 10) or control arm (n=10), and will be identified by a randomly assigned identification number. Among those allocated to the experimental groups, 10 patients with psychotic disorders will be randomly selected to be part of the transitional support group for patients with psychotic disorders, and 10 patients with anxiety or mood disorders will be randomly selected to be part of the transitional self-help group for patients with anxiety or mood disorders. It will be the same for the control group; 5 patients with psychotic disorders will be randomly selected to be part of the control group for patients with psychotic disorders, and 5 patients with anxiety or mood disorders will be randomly selected to be part of the control group for patients with anxiety or mood disorders. In both cases, those who have not been selected will be placed on a substitute list in the event of withdrawal of a selected patient, who will be replaced at random by one of the corresponding substitutes.

The groups will be studied together and separately: the experimental group for patients with psychotic disorders (n=10) will be compared to the control group for patients with psychotic disorders (n=5); the experimental group for patients with anxiety or mood disorders (n=10) will be compared to the control group for patients with anxiety or mood disorders (n=5); and the combined experimental group for patients with psychotic disorders and for patients with anxiety or mood disorders (n=20; 2×10) will be compared to the combined control group for patients with psychotic disorders and anxiety or mood disorders (n=10).

This study will be reported following the CONSORT (Consolidated Standards of Reporting Trials) guidelines [91] and registered before the enrollment of the first participant (eg, ClinicalTrials.gov).

#### Protecting Against Sources of Bias

IUSMM clinical staff will receive no information on how participants scored on the Recovery Assessment Scale and Citizenship Measure questionnaires. PSWs will receive no information on participants’ results related to clinical recovery measures routinely taken for the Signature Bank. The PSWs will be separated from the outpatient clinic therapists and will sign agreements not to discuss cases.

#### Primary and Secondary Outcome Measures

Several instruments have been developed by clinicians and academics to assess clinical recovery. Based on their life narratives and to assess personal-civic recovery, measurement tools have also been developed through community-based participatory research and validated by persons living with MHPIs (eg, the Recovery Assessment Scale and the Citizenship Measure questionnaires). As users of mental health services typically tend to prefer interventions to help them recover, reintegrate with society, and achieve their personal goals [92], we propose this pre-post research feasibility trial design to evaluate the outcomes on personal-civic recovery (primary outcome), on clinical recovery and stress- or anxiety-related responses to the COVID-19 pandemic (secondary outcome).

#### Outcome Measures at Follow-up

The COVID-19 Stress Scales (36 items) and the measures of personal-civic recovery (47 items) will be repeated, along with the following measures of clinical recovery, which are routinely collected among all Signature Bank participants:

1. Anxiety: Anxiety State-Trait Anxiety Inventory Form Y6, 6 items (STAI-Y6) [93];
2. Depression: Depression Patient Health Questionnaire, 9 items (PHQ-9) [94];
3. Alcohol dependence: Alcohol Use Disorders Identification Test, 10 items (AUDIT-10) [95];
4. Drug dependence: Drug Abuse Screening Test, 10 items (DAST-10) [96];
5. Psychosis: Psychosis Screening Questionnaire, 12 items (PSQ) [97]; and
6. Social functioning: WHO Disability Assessment Schedule, 12 items (WHODAS 2.0) [98].

##### The COVID-19 Stress Scales

Research and clinical observations suggest that during times of pandemic many people exhibit stress- or anxiety-related responses that include fear of becoming infected, fear of coming into contact with possibly contaminated objects or surfaces, fear of foreigners who might be carrying infection (ie, disease-related xenophobia), fear of the socioeconomic consequences of the pandemic, compulsive checking of and reassurance seeking related to possible pandemic-related threats, and traumatic stress symptoms about the pandemic (eg, nightmares, intrusive thoughts). Taylor et al developed the 36-item COVID-19 Stress Scales to measure these features, as they pertain to COVID-19. The COVID-19 Stress Scales were developed to better understand and assess COVID-19–related distress. A stable 5-factor solution was identified, corresponding to scales assessing COVID-19–related stress and anxiety symptoms: (1) concerns related to danger and contamination (12 items, Cronbach α=0.94); (2) concerns about economic consequences (6 items, Cronbach α=0.90); (3) xenophobia (6 items, Cronbach α=0.92); (4) traumatic stress symptoms (6 items, Cronbach α=0.93), and (5) compulsive checking and reassurance seeking (6 items, Cronbach α=0.83). In collaboration with the original authors (ie, Steven Taylor), we have translated the COVID-19 Stress Scales into French, and it can be completed using a 5-point Likert scale.

##### The Recovery Assessment Scale

Salzer and Brusilovskiy [99] have published an in-depth review of the quantitative properties of the Recovery Assessment Scale, based on 77 articles that included psychometric data. They concluded that these studies indicate very good results for internal consistency, test-retest reliability, and internal reliability. Among the tools available to empirically assess recovery, this scale has been the most published. Its items cover the following five dimension scales: (1) personal confidence (9 items, Cronbach α=0.86), (2) willingness to ask for help (3 items, Cronbach α=0.83), (3) goal and success orientation (5 items, Cronbach α=0.68), (4) reliance on others (4 items, Cronbach α=0.65), and (5) no domination by symptoms (3 items, Cronbach α=0.73).

##### The Citizenship Measure

The Citizenship Measure was developed through a community-based participatory research design in response to a prompt (*For me, being a citizen means…*) suggested by persons living with MHPIs who were involved as research partners and research staff. The Citizenship Measure items cover the following five dimensions: (1) self-determination (6 items, Cronbach α=0.67), (2) respect by others (4 items, Cronbach α=0.74), (3) involvement in the community (4 items, Cronbach α=0.65), (4) basic needs (5 items, Cronbach α=0.60), and (5) access to services (4 items, Cronbach α=0.60).

When completing the Recovery Assessment Scale and Citizenship Measure questionnaires, participants will be asked to rate on a 5-point Likert scale (1=strongly disagree, 5=strongly agree) the extent to which the respective statements apply to them since the COVID-19 pandemic (in French: *situation depuis le début de la période COVID-19*). To simplify the instructions, COVID-19 will be referred to as “the virus.” Although COVID-19 actually refers to the disease and SARS-CoV-2 is the virus, in line with the developers of the COVID-19 Stress Scales, we expect that many respondents will not be aware of this distinction. Based on feedback from the pilot testing, respondents readily understood what the developers of these scales were referring to.

#### Recruitment

As of February 2020, 2136 IUSMM patients have been enrolled in the Signature Bank since the year 2012, including 822 individuals with psychotic disorders, and 853 with anxiety and/or mood disorders. The study was approved by the local ethics committee in accordance with the Declaration of Helsinki, and the Signature Bank’s management framework provides further details on recruitment and consent forms. Between August 26, 2019, and February 26, 2020 (6 months), a preliminary validation study [100] allowed us to recruit 93 of the Signature Bank participants diagnosed with either psychotic disorders, or with anxiety and mood disorders. In total, 36 were female (39%) and 57 were male (61%). They further completed the Recovery Assessment Scale and Citizenship Measure, both of which a 5-point Likert scale was used to rate items. For the former, the mean was 3.77 (out of 5; SD 0.78). For the latter, the mean was 3.91 (out of 5; SD 0.63). The type of participants we need can thus easily complete our questionnaires in parallel to those of the Signature Bank. A research assistant with lived experience of an MHPI will contact by phone all the above-mentioned 93 Signature Bank participants who have already been in touch for the previous validation study and who have already accepted to be contacted again by our team for such purposes. If needed, other Signature Bank participants who meet the inclusion criteria will also be contacted until 30 Information and Consent forms are returned. The Information and Consent form will be sent by email to those who provide an email address. They will be offered CA$20 as compensation indemnity for the baseline and follow-up completions of the Recovery Assessment Scale, the Citizenship Measure, and the Bem Sex Role Inventory (see the *Sex and Gender* section).

#### Quantitative Analyzes

Baseline characteristics will be summarized, including for the measures of clinical recovery (see the *Outcome Measures at Follow-up* section), the Recovery Assessment Scale, the Citizenship Measure (personal-civic recovery), the COVID-19 Stress Scales, and the Bem Sex Role Inventory. All participants with data at baseline (T1) and follow-up will be included in the analyses. We will compare within-patient change from baseline to intervention versus control group in the study outcome measures using the Student paired sample *t* test. The analysis will run on intervention and control practices separately so as to explore practice level impact on the differences in outcomes. Comparison of confidence interval and effect size between groups will be assessed.

#### Qualitative Analyses

Each workshop and the corresponding debriefing session among PSWs will be transcribed verbatim. The data analysis team will employ thematic analysis [101-103], and a combined deductive and inductive approach to coding [104]. We are particularly interested in understanding life trajectories and transitions in relation to the five domains of the Recovery Assessment Scale and the five clusters of the Citizenship Measure. The chosen approach combines the perspective of the life course and the relational perspective of social networks. This approach makes it possible to understand the different trajectories that make up the life course of individuals in the light of the dynamic relationships that place them (or not) in support networks while taking into account their subjectivity and ability to act (agency). From the life course perspective, the development of the person is posited as a process that does not necessarily stop at predetermined stages [105]. The structured and unstructured material will also be analyzed through natural language processing [106], which is one area of artificial intelligence using computational linguistics that provides parsing and semantic interpretation of text, which allows systems to learn, analyze, and understand human language.

#### Sex and Gender

Biological sex is a categorical construct comprised of genes, anatomy, gonads, and hormones that make up male and female differences [107]. Beyond one’s birth-assigned sex, sociocultural gender refers to diverse roles, orientations, and identities that influence health across lifespan development. Sex and gender have rarely been considered together in recovery research. To address this gap, we will conduct secondary analyses stratified by sex following recent recommendations in promoting rigor and reproducibility in health research [108]. In qualitative analyses, verbatims will also be coded with considerations of participants’ sex in order to identify themes unique to men and women. For example, social support is a health-promoting factor that is experienced differently by men and women, as we have seen in a previous study with the results of the personal-civic recovery measures. In addition to sex, we will explore sociocultural gender roles using a validated questionnaire (Bem Sex Role Inventory) that will allow us to assess dimensions of masculinity, femininity, and androgyny along continuums [109,110]. Previous work by Juster et al [111] has shown how measuring gender-roles provides unique within-sex understanding of mental and physical health. In quantitative analyses, we will explore correlations among gender roles (masculine and feminine subscales) in association with study outcomes representing recovery. Taken together, our sex- and gender-based analyses will provide insights into sex-specific and gender-specific recovery.

### Ethical Considerations

Declaration of Helsinki protocols are being followed, and patients will give written informed consent. The study was approved on June 16, 2020, by the Research Ethics Committees of the Montreal Mental Health University Institute (#2020-1948). For all participants of the Signature Bank, including those participating in the research presented in this manuscript, an overseeing mental health expert have ruled that all adult patients were deemed ethically and medically capable of consenting for their participation.

## Results

This pilot study was funded in March 2020. Recruitment took place during the months of July and August 2020. Table 1 presents the distribution of recruited participants. We anticipate the publication of two key papers in accordance with the Canadian Institutes of Health Research open access policy: (1) the registered research protocol and (2) description of the main conclusions of our case-control study. Table 2 presents the calendar of activities.

**Table 1:** Distributions of participants in the pilot study.

Characteristic		Participants, n (%)
**Total eligible participants (N=92)**		
	Participants with AMD^a^	31 (33.7)
	Participants with PD^b^	61 (66.3)
**Total eligible participants who agreed to receive the ICF^c^ by email (N=92)**		54 (58.7)
	Participants with AMD (n=31)	23 (74.2)
	Participants with PD (n=61)	31 (50.8)
**Total eligible participants who returned the signed ICF (n=54)**		36 (66.7)
	Participants with AMD (n=23)	17 (73.9)
	Participants with PD (n=31)	19 (61.3)
**Total eligible participants who completed the questionnaires at T1 (n=36)**		32 (88.8)
	Participants with AMD (n=17)	16 (94.1)
	Participants with PD (n=19)	19 (100.0)
**Total eligible participants who completed the questionnaires at T2 (n=30)**		24 (80.0)
	Participants with AMD in the experimental group (n=10)	7 (70.0)
	Participants with PD in the experimental group (n=10)	10 (100.0)
	Participants with AMD or PD in the control group (n=10)	7 (70.0)

^a^AMD: anxiety and mood disorders

^b^PD: psychotic disorders.

^c^ICF: Information and Consent Form.

**Table 2:** Calendar of activities.

Date	Activity	
	Convergent and Concurrent Validity Between Clinical Recovery and Personal-Civic Recovery	Effects of Online and Recovery-Oriented Peer Support Groups Facilitated by Peer Support Workers
	ClinicalTrials.gov ID NCT04125030	ClinicalTrials.gov ID NCT04445324
September 2019 to March 2020	Study suspended due to the COVID-19 pandemic 92 eligible participants completed the study	—^a^
April-May 2020	—	Writing and submission of the research protocol to the institutional review board
June 2020	—	Institutional review board approval of research protocol
July 2020	Submission of the study protocol for publication in a peer-reviewed journal	Submission of the study protocol for publication in a peer-reviewed journal
August 2020	—	Recruitment of study participants among those 92 from the previous validation study Precompletion of measures (T1)
September to October 2020	Publication of the research protocol [100]	10 weekly peer support groups for patients with psychotic disorders 10 weekly peer support groups for patients with anxiety or mood disorders
November 2020	Data analyses	Postcompletion of measures (T2) Addenda to study protocol Submission of the revised study protocol for publication
December 2020	Submission of the study’s main conclusions for publication in an open access peer-reviewed journal	Data analyses Submission of the study’s main conclusions for publication in an open access peer-reviewed journal

^a^Not applicable.

## Discussion

Qualitative and quantitative results will be provided to all stakeholders and knowledge users, and posted on our massive open online course (MOOC) platform. Several PSWs and engaged service users take turns as teaching partners in *The Fundamentals of Recovery* MOOC [112], for which there are 1553 registrants from 51 different countries (Multimedia Appendix 1).

MOOCs are free interactive step-by-step courses developed by universities with the aim of reaching an unlimited number of participants and to create a community of lifelong e-learners (electronic learners). PSWs will be involved in the presentation and discussion of the findings, and acknowledged as coauthors in the publications, whenever appropriate, including within the MOOC (second edition). Indeed, the MOOC will be used as a knowledge translation platform for ongoing discussion among registrants, and updated with the findings of this pilot study in particular. Undoubtedly, the COVID-19 pandemic has disrupted many aspects of academic medical missions [113]. When the pandemic has subsided, although there will likely be some “return to normal,” some of the innovations developed in response to the COVID-19 pandemic will most likely remain a part of academic psychiatry’s everyday clinical and educational operations. The MOOC was not originally conceived as an innovative response to any particular pandemic. It has been nevertheless particularly appreciated in times of lockdown due to the COVID-19 pandemic. For the first 20 weeks of 2020, Figure 1 shows the cumulative total of registrants, whereas Figure 2 shows the weekly variations of new registrants to the MOOC; both figures show accelerated counts since WHO officials announced on March 11, 2020, that the COVID-19 contagion should henceforth be considered a pandemic.

**Figure 1:**
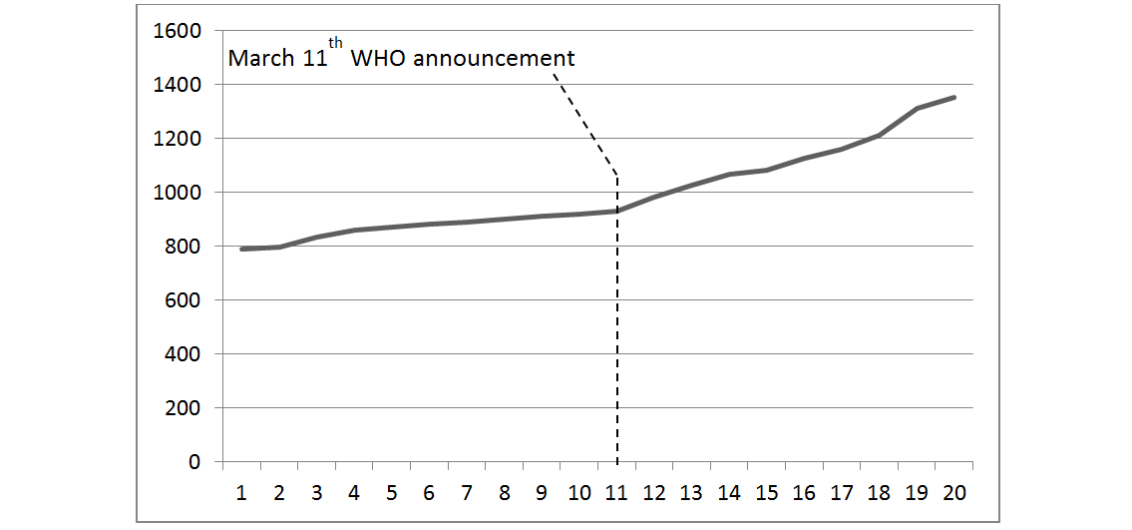
Cumulative total of MOOC (massive open online course) registrants during the first 20 weeks of 2020. WHO: World Health Organization.

**Figure 2:**
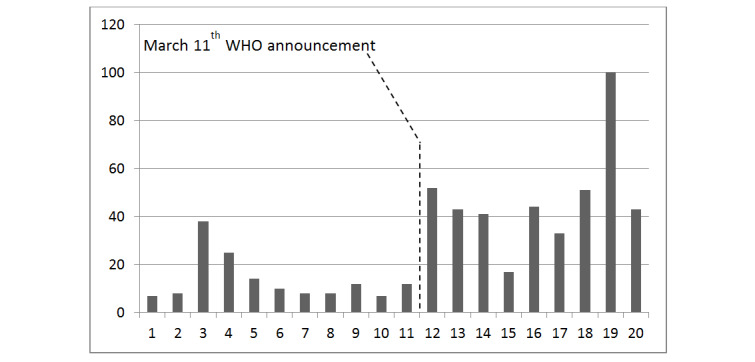
Weekly variations in new registrants to the MOOC (massive open online course) during the first 20 weeks of 2020. WHO: World Health Organization.

In 2017, before the current COVID-19 crisis, Bendezu-Quispe and colleagues [114] suggested that the emergence and re-emergence of communicable infectious diseases like Ebola or Zika were already increasing the necessity of knowledgeable and skilled health professionals, specifically with MOOCs arising as opportunities that allow people around the world to participate in higher-education courses. They argued that MOOCs can be used to learn about health issues of global relevance, and with the necessity of fast divulgation of knowledge and skills. Given the scope of mental health problems and the constraints of resources in training psychiatrists and other mental health professionals in most parts of the world, continuing education for medical and health or social services professionals and students, including in primary care, is vital [115]. In this MOOC, PSWs are included as training partners, as well as a subgroup of colearners.

Moreover, millions of people worldwide experienced moderate to severe levels of stress- or anxiety-related symptoms in response to COVID-19. This is true for the general population [116] as well as for people already diagnosed with psychotic disorders [117] or anxiety and mood disorders [118]. Yet, if general conditions remain the same for the participants of the experimental groups and for those of the control groups, and significantly different benefits are still observed among these groups, these differences will, in principle, be attributable to the intervention and not to the pandemic context.

Some psychiatrists even warn of a “tsunami” of mental illness due to problems arising during lockdown. They are particularly concerned that children and older adults are not receiving the support they need because of school closures, self-isolation, and fear of hospitals [119]. It could take years for some people to recover from these problems and find their own recovery pathway if they do not get support. During the pandemic, our new online intervention, combined with the MOOC, will add to already existing services. Several studies report on such experiments, such as online peer support for patients [120] but also for health professionals on the frontline of the COVID-19 outbreak [121] and whose mental health and well-being are at stake. This pandemic context indeed has major impacts on stress, anxiety, and possible depression in people who are already in treatment [122] as well as the general population and current health care workers, including PSWs. Supporting the mental health of medical staff and affiliated health care workers is a critical part of the public health response [123]. Given that three Integrated University Health and Social Services Centres affiliated with the University of Montreal—with a total of more than 40,000 employees—are supporting this pilot project, they may wish to come together and widen access to this relatively low-cost intervention for other current patients as well as their employees in need of support and information to prevent deterioration of their own mental health and recovery potential. The arrival of PSWs in the workforce as a new type of additional provider will no doubt be welcome.

Beyond the current acute context, this feasibility study of a trial and corresponding future RCT, plus the MOOC as an innovative knowledge translation strategy, have the potential to demonstrate the relevance of this online group intervention of PSWs for many more current and future patients. Indeed, the Quebec Ministerial Mental Health Action Plan currently in force mentions the Assertive Community Treatment teams as potential models for the inclusion PSWs. Several reviews concluded that Assertive Community Treatment is more effective than standard services in reducing hospital use and increasing community tenure, and numerous practice guidelines endorsed this model as an evidence-based practice for the treatment of psychotic disorders like schizophrenia [124]. Psychotic disorders affect about 1% of the population, while anxiety and mood disorders are much more common disorders from which 10% of the population experiences during normal times, a figure that will inevitably rise with the current COVID-19 pandemic and its aftermaths. There are already existing (online) self-help groups for these people, with or without PSWs, but which are not necessarily complementary to treatment offered by formal public mental health services, as is the case for people treated by Assertive Community Treatments teams where there are PSWs. Conversely, as the potential of digital mental health has become urgently apparent, the surge in interest and use of digital health to meet the demands of patients in quarantine, with social and physical distancing restrictions, and a lack of in-person care has centered on anxiety and mood disorders—and largely ignored those with psychotic disorders [120]. As uses of telehealth during the COVID-19 crisis increase, the potential of digital mental health to increase access is becoming clearer [125,126]. Access to a transitional and intermediary online self-help groups between the institutional environment and the community environment, for both people living with psychotic disorders or anxiety and mood disorders alike, could become a good practice to recommend beyond the current context of the COVID-19 pandemic.

## Appendix

Multimedia Appendix 1 Profile of MOOC registrants after the first year (N=1553).

## Abbreviations

AUDIT-10: Alcohol Use Disorders Identification Test, 10 items
CanMEDS: Canadian Medical Educational Directives for Specialists
CONSORT: Consolidated Standards of Reporting Trials
DAST-10: Drug Abuse Screening Test, 10 items
e-learner: electronic learner
ICD-10: International Classification of Disease–10th Revision
IUSMM: Institut universitaire en santé mentale de Montréal
MHPI: mental health problems or illness
MOOC: massive open online course
PHQ-9: Patient Health Questionnaire, 9 items
PSQ: Psychosis Screening Questionnaire, 12 items
PSW: peer support worker
RCT: randomized controlled trial
STAI-Y6: Anxiety State-Trait Anxiety Inventory Form Y6, 6 items
WHO: World Health Organization
WHODAS 2.0: WHO Disability Assessment Schedule, 12 items
