# Supplementary material for: Online and Recovery-Oriented Support Groups Facilitated by Peer Support Workers in Times of COVID-19: Protocol for a Feasibility Pre-Post Study
Source: JMIR Res Protoc. 2020 Dec 18;9(12):e22500. doi: 10.2196/22500 (PMC7752185; doi:10.2196/22500)
Supplement: Multimedia Appendix 1 [file resprot_v9i12e22500_app1.docx]

Profile of Massive Open Online Course (MOOC) registrants after first year (N=1553).

| **Age** | |
| --- | --- |
| Median age | 38 |
| ≤25 years old | 12.7% |
| 26-40 years old | 44.6% |
| ≥41 years old | 42.7% |
|  |  |
| **Sex** | |
| Females | 68.8% |
| Males  Other | 30.4%  0.8% |
|  |  |
| **Education** | |
| High school | 11.1% |
| College degree | 36.7% |
| Master or PhD degree | 48.5% |
| Other | 3.7% |
|  |  |
| **Location** |  |
| Canada | 42.7% |
| France | 41.7% |
| Morocco | 3.0% |
| Haiti | 2.0% |
| Other | 11.2% |
